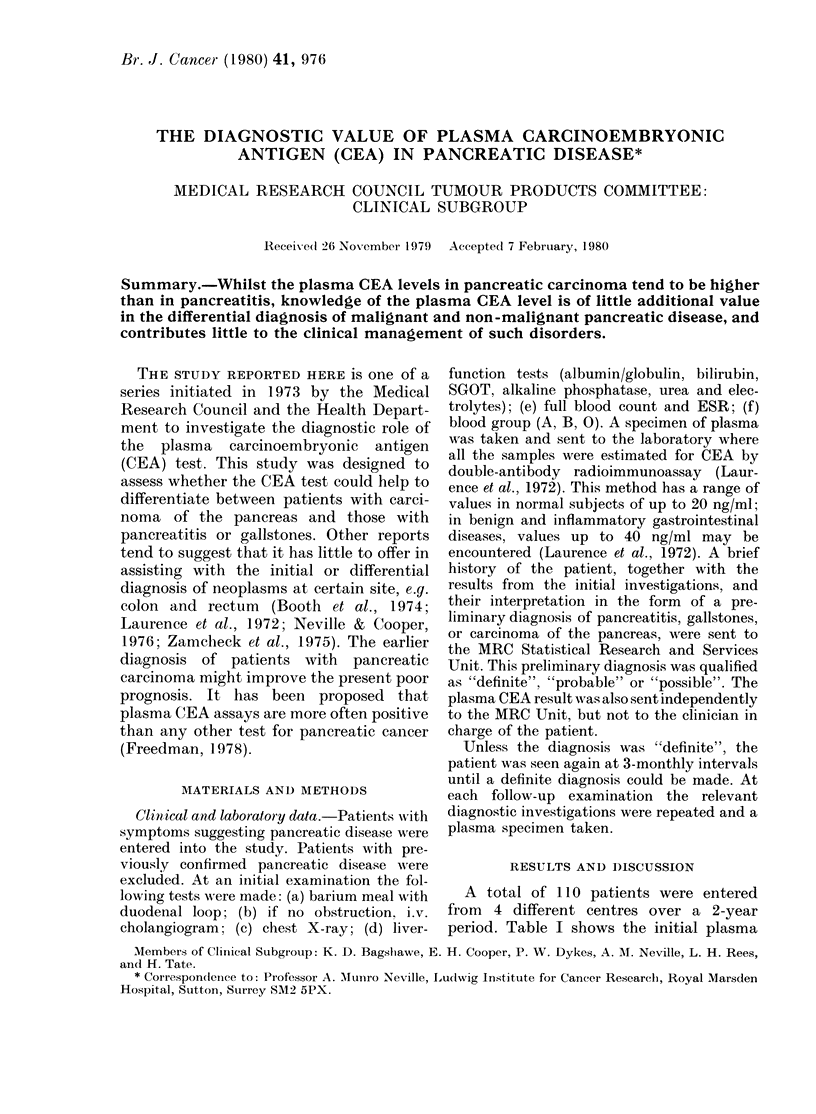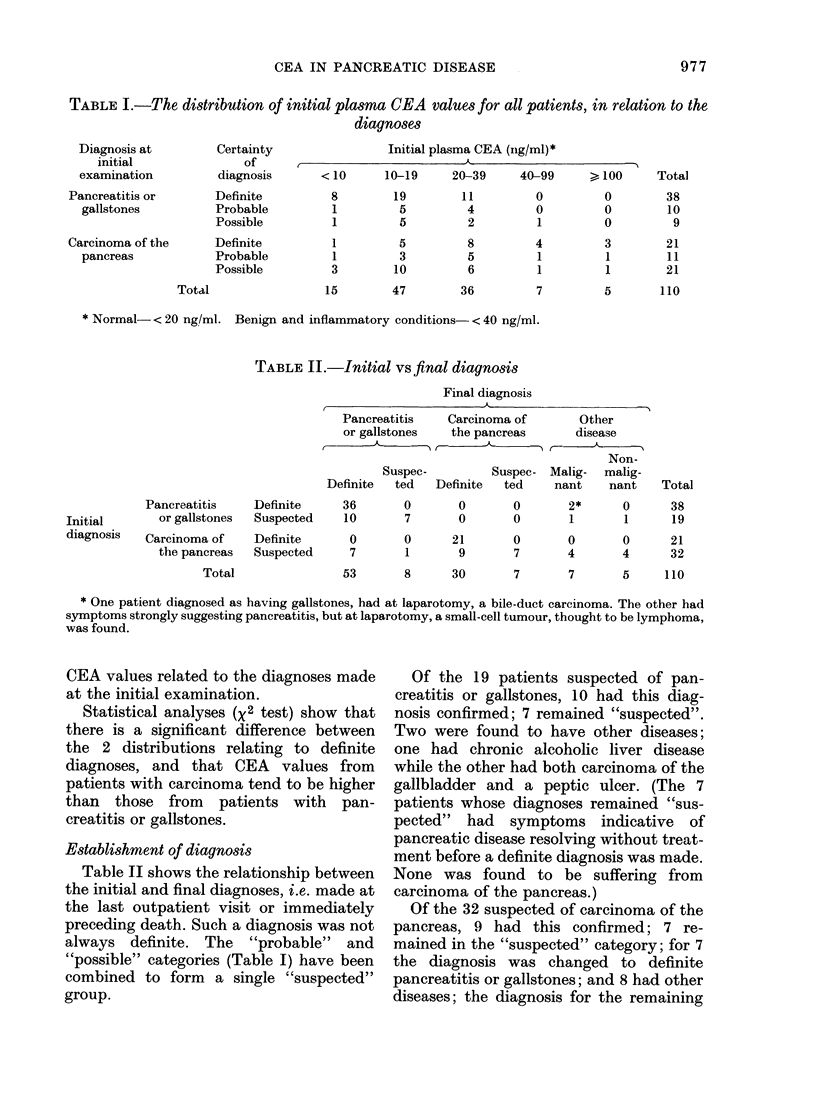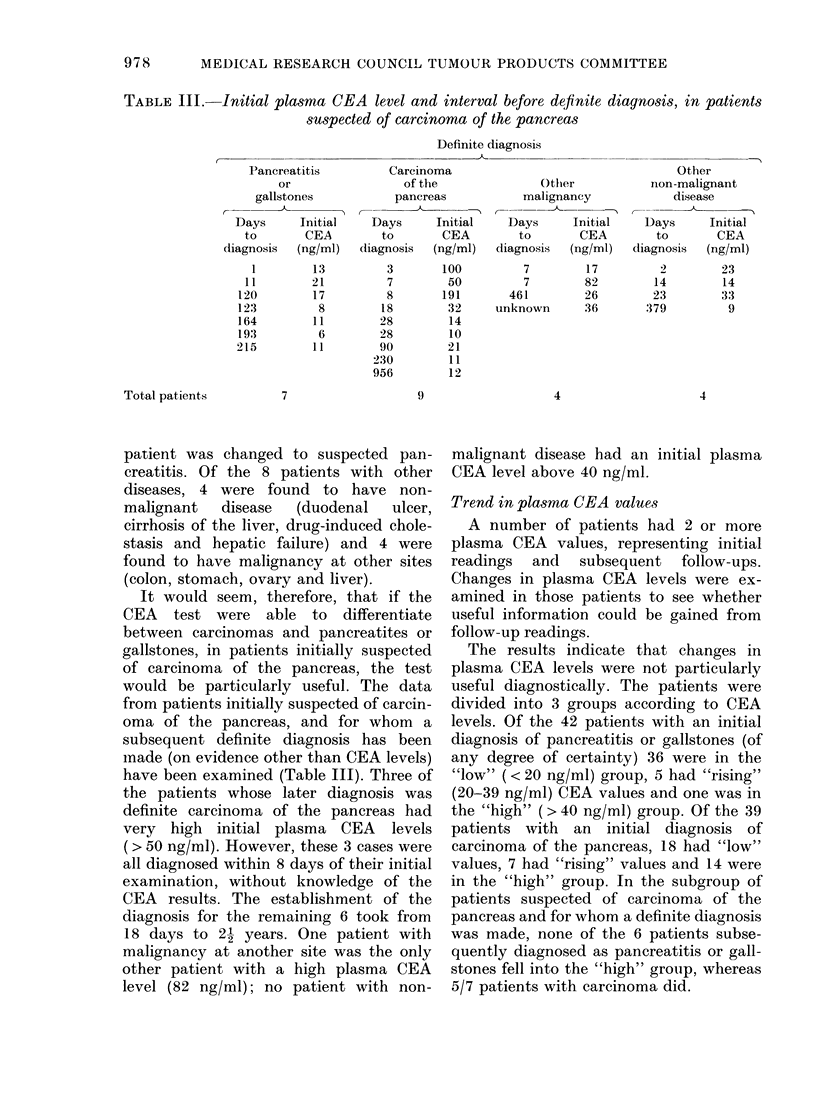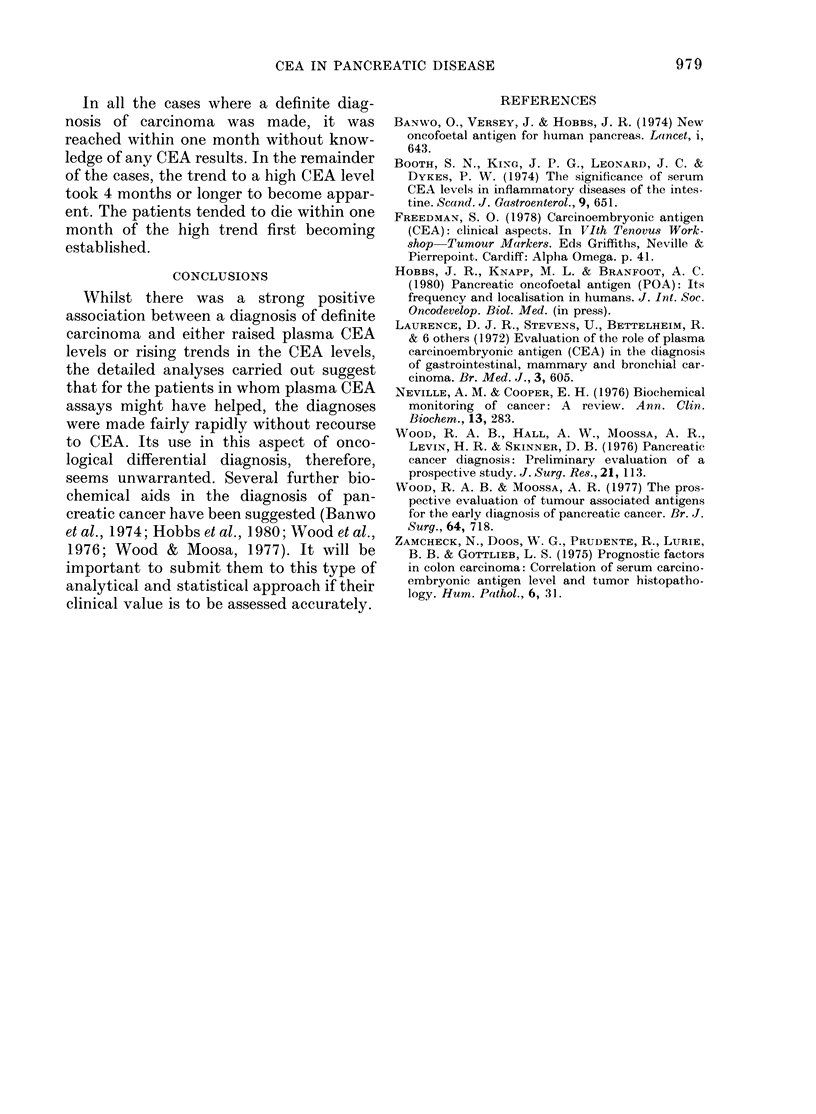# The diagnostic value of plasma carcinoembryonic antigen (CEA) in pancreatic disease. Medical Research Council Tumour Products Committee: Clinical Subgroup.

**DOI:** 10.1038/bjc.1980.176

**Published:** 1980-06

**Authors:** 

## Abstract

Whilst the plasma CEA levels in pancreatic carcinoma tend to be higher than in pancreatitis, knowledge of the plasma CEA level is of little additional value in the differential diagnosis of malignant and non-malignant pancreatic disease, and contributes little to the clinical management of such disorders.


Br. J. Cancer (1980) 41, 976

### THE DIAGNOSTIC VALUE OF PLASMA CARCINOEMBRYONIC ANTIGEN (CEA) IN PANCREATIC DISEASE*

MEDICAL RESEARCH COUNCIL TUMOUR PRODUCTS COMMITTEE: CLINICAL SUBGROUP

Received 26 November 1979 Accepted 7 February, 1980

**Summary.—Whilst the plasma CEA levels in pancreatic carcinoma tend to be higher than in pancreatitis, knowledge of the plasma CEA level is of little additional value in the differential diagnosis of malignant and non-malignant pancreatic disease, and contributes little to the clinical management of such disorders.**

THE STUDY REPORTED HERE is one of a series initiated in 1973 by the Medical Research Council and the Health Department to investigate the diagnostic role of the plasma carcinoembryonic antigen (CEA) test. This study was designed to assess whether the CEA test could help to differentiate between patients with carcinoma of the pancreas and those with pancreatitis or gallstones. Other reports tend to suggest that it has little to offer in assisting with the initial or differential diagnosis of neoplasms at certain site, *e.g.* colon and rectum (Booth *et al.*, 1974; Laurence *et al.*, 1972; Neville & Cooper, 1976; Zamcheck *et al.*, 1975). The earlier diagnosis of patients with pancreatic carcinoma might improve the present poor prognosis. It has been proposed that plasma CEA assays are more often positive than any other test for pancreatic cancer (Freedman, 1978).

### MATERIALS AND METHODS

*Clinical and laboratory data.*—Patients with symptoms suggesting pancreatic disease were entered into the study. Patients with previously confirmed pancreatic disease were excluded. At an initial examination the following tests were made: (a) barium meal with duodenal loop; (b) if no obstruction, i.v. cholangiogram; (c) chest X-ray; (d) liver-function tests (albumin/globulin, bilirubin, SGOT, alkaline phosphatase, urea and electrolytes); (e) full blood count and ESR; (f) blood group (A, B, O). A specimen of plasma was taken and sent to the laboratory where all the samples were estimated for CEA by double-antibody radioimmunoassay (Laurence *et al.*, 1972). This method has a range of values in normal subjects of up to 20 ng/ml; in benign and inflammatory gastrointestinal diseases, values up to 40 ng/ml may be encountered (Laurence *et al.*, 1972). A brief history of the patient, together with the results from the initial investigations, and their interpretation in the form of a preliminary diagnosis of pancreatitis, gallstones, or carcinoma of the pancreas, were sent to the MRC Statistical Research and Services Unit. This preliminary diagnosis was qualified as "definite", "probable" or "possible". The plasma CEA result was also sent independently to the MRC Unit, but not to the clinician in charge of the patient.

Unless the diagnosis was "definite", the patient was seen again at 3-monthly intervals until a definite diagnosis could be made. At each follow-up examination the relevant diagnostic investigations were repeated and a plasma specimen taken.

### RESULTS AND DISCUSSION

A total of 110 patients were entered from 4 different centres over a 2-year period. Table I shows the initial plasma

Members of Clinical Subgroup: K. D. Bagshawe, E. H. Cooper, P. W. Dykes, A. M. Neville, L. H. Rees, and H. Tate.
* Correspondence to: Professor A. Munro Neville, Ludwig Institute for Cancer Research, Royal Marsden Hospital, Sutton, Surrey SM2 5PX.

TABLE I.—*The distribution of initial plasma CEA values for all patients, in relation to the diagnoses*

| Diagnosis at initial examination | Certainty of diagnosis | Initial plasma CEA (ng/ml)* <10 | 10–19 | 20–39 | 40–99 | ≥100 | Total |
|---|---|---|---|---|---|---|---|
| Pancreatitis or gallstones | Definite | 8 | 19 | 11 | 0 | 0 | 38 |
| | Probable | 1 | 5 | 4 | 0 | 0 | 10 |
| | Possible | 1 | 5 | 2 | 1 | 0 | 9 |
| Carcinoma of the pancreas | Definite | 1 | 5 | 8 | 4 | 3 | 21 |
| | Probable | 1 | 3 | 5 | 1 | 1 | 11 |
| | Possible | 3 | 10 | 6 | 1 | 1 | 21 |
| Total | | 15 | 47 | 36 | 7 | 5 | 110 |

* Normal—<20 ng/ml. Benign and inflammatory conditions—<40 ng/ml.

TABLE II.—*Initial* vs *final diagnosis*

| | | | Final diagnosis: Pancreatitis or gallstones | | Carcinoma of the pancreas | | Other disease | | |
|---|---|---|---|---|---|---|---|---|---|
| | | | Definite | Suspected | Definite | Suspected | Malignant | Non-malignant | Total |
| Initial diagnosis | Pancreatitis or gallstones | Definite | 36 | 0 | 0 | 0 | 2* | 0 | 38 |
| | | Suspected | 10 | 7 | 0 | 0 | 1 | 1 | 19 |
| | Carcinoma of the pancreas | Definite | 0 | 0 | 21 | 0 | 0 | 0 | 21 |
| | | Suspected | 7 | 1 | 9 | 7 | 4 | 4 | 32 |
| | Total | | 53 | 8 | 30 | 7 | 7 | 5 | 110 |

* One patient diagnosed as having gallstones, had at laparotomy, a bile-duct carcinoma. The other had symptoms strongly suggesting pancreatitis, but at laparotomy, a small-cell tumour, thought to be lymphoma, was found.

CEA values related to the diagnoses made at the initial examination.

Statistical analyses ($\chi^2$ test) show that there is a significant difference between the 2 distributions relating to definite diagnoses, and that CEA values from patients with carcinoma tend to be higher than those from patients with pancreatitis or gallstones.

*Establishment of diagnosis*

Table II shows the relationship between the initial and final diagnoses, *i.e.* made at the last outpatient visit or immediately preceding death. Such a diagnosis was not always definite. The "probable" and "possible" categories (Table I) have been combined to form a single "suspected" group.

Of the 19 patients suspected of pancreatitis or gallstones, 10 had this diagnosis confirmed; 7 remained "suspected". Two were found to have other diseases; one had chronic alcoholic liver disease while the other had both carcinoma of the gallbladder and a peptic ulcer. (The 7 patients whose diagnoses remained "suspected" had symptoms indicative of pancreatic disease resolving without treatment before a definite diagnosis was made. None was found to be suffering from carcinoma of the pancreas.)

Of the 32 suspected of carcinoma of the pancreas, 9 had this confirmed; 7 remained in the "suspected" category; for 7 the diagnosis was changed to definite pancreatitis or gallstones; and 8 had other diseases; the diagnosis for the remaining

TABLE III.—*Initial plasma CEA level and interval before definite diagnosis, in patients suspected of carcinoma of the pancreas*

| | Definite diagnosis | | | | | | | |
|---|---|---|---|---|---|---|---|---|
| | Pancreatitis or gallstones | | Carcinoma of the pancreas | | Other malignancy | | Other non-malignant disease | |
| | Days to diagnosis | Initial CEA (ng/ml) | Days to diagnosis | Initial CEA (ng/ml) | Days to diagnosis | Initial CEA (ng/ml) | Days to diagnosis | Initial CEA (ng/ml) |
| | 1 | 13 | 3 | 100 | 7 | 17 | 2 | 23 |
| | 11 | 21 | 7 | 50 | 7 | 82 | 14 | 14 |
| | 120 | 17 | 8 | 191 | 461 | 26 | 23 | 33 |
| | 123 | 8 | 18 | 32 | unknown | 36 | 379 | 9 |
| | 164 | 11 | 28 | 14 | | | | |
| | 193 | 6 | 28 | 10 | | | | |
| | 215 | 11 | 90 | 21 | | | | |
| | | | 230 | 11 | | | | |
| | | | 956 | 12 | | | | |
| Total patients | 7 | | 9 | | 4 | | 4 | |

patient was changed to suspected pancreatitis. Of the 8 patients with other diseases, 4 were found to have non-malignant disease (duodenal ulcer, cirrhosis of the liver, drug-induced cholestasis and hepatic failure) and 4 were found to have malignancy at other sites (colon, stomach, ovary and liver).

It would seem, therefore, that if the CEA test were able to differentiate between carcinomas and pancreatites or gallstones, in patients initially suspected of carcinoma of the pancreas, the test would be particularly useful. The data from patients initially suspected of carcinoma of the pancreas, and for whom a subsequent definite diagnosis has been made (on evidence other than CEA levels) have been examined (Table III). Three of the patients whose later diagnosis was definite carcinoma of the pancreas had very high initial plasma CEA levels ($>50$ ng/ml). However, these 3 cases were all diagnosed within 8 days of their initial examination, without knowledge of the CEA results. The establishment of the diagnosis for the remaining 6 took from 18 days to $2\frac{1}{2}$ years. One patient with malignancy at another site was the only other patient with a high plasma CEA level (82 ng/ml); no patient with non-malignant disease had an initial plasma CEA level above 40 ng/ml.

*Trend in plasma CEA values*

A number of patients had 2 or more plasma CEA values, representing initial readings and subsequent follow-ups. Changes in plasma CEA levels were examined in those patients to see whether useful information could be gained from follow-up readings.

The results indicate that changes in plasma CEA levels were not particularly useful diagnostically. The patients were divided into 3 groups according to CEA levels. Of the 42 patients with an initial diagnosis of pancreatitis or gallstones (of any degree of certainty) 36 were in the "low" ($<20$ ng/ml) group, 5 had "rising" (20–39 ng/ml) CEA values and one was in the "high" ($>40$ ng/ml) group. Of the 39 patients with an initial diagnosis of carcinoma of the pancreas, 18 had "low" values, 7 had "rising" values and 14 were in the "high" group. In the subgroup of patients suspected of carcinoma of the pancreas and for whom a definite diagnosis was made, none of the 6 patients subsequently diagnosed as pancreatitis or gallstones fell into the "high" group, whereas 5/7 patients with carcinoma did.

In all the cases where a definite diagnosis of carcinoma was made, it was reached within one month without knowledge of any CEA results. In the remainder of the cases, the trend to a high CEA level took 4 months or longer to become apparent. The patients tended to die within one month of the high trend first becoming established.

### CONCLUSIONS

Whilst there was a strong positive association between a diagnosis of definite carcinoma and either raised plasma CEA levels or rising trends in the CEA levels, the detailed analyses carried out suggest that for the patients in whom plasma CEA assays might have helped, the diagnoses were made fairly rapidly without recourse to CEA. Its use in this aspect of oncological differential diagnosis, therefore, seems unwarranted. Several further biochemical aids in the diagnosis of pancreatic cancer have been suggested (Banwo *et al.*, 1974; Hobbs *et al.*, 1980; Wood *et al.*, 1976; Wood & Moosa, 1977). It will be important to submit them to this type of analytical and statistical approach if their clinical value is to be assessed accurately.

### REFERENCES

Banwo, O., Versey, J. & Hobbs, J. R. (1974) New oncofoetal antigen for human pancreas. *Lancet*, i, 643.

Booth, S. N., King, J. P. G., Leonard, J. C. & Dykes, P. W. (1974) The significance of serum CEA levels in inflammatory diseases of the intestine. *Scand. J. Gastroenterol.*, **9**, 651.

Freedman, S. O. (1978) Carcinoembryonic antigen (CEA): clinical aspects. In *VIth Tenovus Workshop—Tumour Markers.* Eds Griffiths, Neville & Pierrepoint. Cardiff: Alpha Omega. p. 41.

Hobbs, J. R., Knapp, M. L. & Branfoot, A. C. (1980) Pancreatic oncofoetal antigen (POA): Its frequency and localisation in humans. *J. Int. Soc. Oncodevelop. Biol. Med.* (in press).

Laurence, D. J. R., Stevens, U., Bettelheim, R. & 6 others (1972) Evaluation of the role of plasma carcinoembryonic antigen (CEA) in the diagnosis of gastrointestinal, mammary and bronchial carcinoma. *Br. Med. J.*, **3**, 605.

Neville, A. M. & Cooper, E. H. (1976) Biochemical monitoring of cancer: A review. *Ann. Clin. Biochem.*, **13**, 283.

Wood, R. A. B., Hall, A. W., Moossa, A. R., Levin, H. R. & Skinner, D. B. (1976) Pancreatic cancer diagnosis: Preliminary evaluation of a prospective study. *J. Surg. Res.*, **21**, 113.

Wood, R. A. B. & Moossa, A. R. (1977) The prospective evaluation of tumour associated antigens for the early diagnosis of pancreatic cancer. *Br. J. Surg.*, **64**, 718.

Zamcheck, N., Doos, W. G., Prudente, R., Lurie, B. B. & Gottlieb, L. S. (1975) Prognostic factors in colon carcinoma: Correlation of serum carcinoembryonic antigen level and tumor histopathology. *Hum. Pathol.*, **6**, 31.